# Kleine–Levin syndrome: Etiology, diagnosis, and treatment

**DOI:** 10.4103/0972-2327.74185

**Published:** 2010

**Authors:** Santosh Ramdurg

**Affiliations:** Department of Psychiatry, All India Institute of Medical Sciences, Ansari Nagar, New Delhi 110 029, India

**Keywords:** Hypersomnia, hypersexuality, Kleine–Levin syndrome, megaphagia, periodic

## Abstract

Kleine–Levin syndrome (KLS) is a rare sleep disorder mainly affecting teenage boys in which the main features are intermittent hypersomnolence, behavioral and cognitive disturbances, hyperphagia, and in some cases hypersexuality. Each episode is of brief duration varying from a week to 1–2 months and affected people are entirely asymptomatic between episodes. No definite cause has been identified, and no effective treatments are available even though illness is having well-defined clinical features. Multiple relapses occur every few weeks or months, and the condition may last for a decade or more before spontaneous resolution. In this study, PubMed was searched and appropriate articles were reviewed to highlight etiology, clinical features, and management of KLS. On the basis of this knowledge, practical information is offered to help clinicians about how to investigate a case of KLS, and what are the possible treatment modalities available currently for the treatment during an episode and interepisodic period for prophylaxis. Comprehensive research into the etiology, pathophysiology, investigation, and treatments are required to aid the development of disease-specific targeted therapies.

## Introduction

Kleine–Levin syndrome (KLS) is a rare disease characterized by recurrent episodes of hypersomnia and to various degrees, behavioral or cognitive disturbances, compulsive eating behavior, and hypersexuality.[1] The disease predominantly affects adolescent males. Although no population-based studies reporting on KLS prevalence are available, it is generally considered an exceptionally rare disease. What appears to be the first case of KLS was reported by Brierre de Boismont in 1862. It is notable that this case occurred several decades prior to the 1916–1927 epidemic of encephalitis lethargic. Multiple cases of recurrent hypersomnia were first collected and reported in Frankfurt by Kleine.[2] Levin emphasized the association of periodic somnolence with morbid hunger in 1929 and 1936.[3,4] Critchley reviewed 15 previously published cases, added 11 of his own personal cases, notably young marines in the British Royal Navy where he had served during World War II and gave the eponymous name to the disease, “Kleine–Levin syndrome.”[5] Kleine–Levin syndrome was further defined by Schmidt in 1990, who established the following diagnostic criteria: (1) predominance in adolescent males, (2) onset in adolescence, (3) periodic hypersomnia, (4) hyper/mega/polyphagia, (5) associated behavioral and psychological changes, benign clinical course with spontaneous disappearance of clinical symptoms, and (6) lack of other neurological or psychiatric diseases. In 1990, the diagnostic criteria for Kleine–Levin syndrome were modified in the International Classification of Sleep Disorders, where it was defined as a syndrome composed of recurring episodes of undue sleepiness lasting some days, which may or may not be associated with hyperphagia and abnormal behavior (ICSD 1990).[6] The KLS has been classified into primary and secondary depending on identifiable underlying organic causes for recurrent hypersomnia and other behavioral symptoms.

## Epidemiology

The exact prevalence of KLS is unknown, but it is considered a very rare disease, possibly affecting one in a million. The literature search by Arnulf *et al*. in PubMed between 1962 and 2004 in English and non-English languages found 186 cases.[7] The cases were reported worldwide. Majority cases were reported in western countries, among one-sixth of patients were found in Israel, suggesting that Jewish heritage could provide a vulnerability for the disease.[8] Men were more frequently affected than women, with a gender ratio of 2:1.[7] The disease affects mostly teenagers. In one meta-analysis, the median age at disease onset was 15 years (range, 4–80 years), with 81% of the cases beginning during the second decade.[9] Cases starting before puberty were rare. Most cases are sporadic, but in few multiplex KLS families have been reported.[10,11] When defining as secondary, the KLS patients having neurologic symptoms before disease onset that persisted during the intervals free of KLS symptoms. The investigation of 186 cases of KLS reported till 2004 found that 18 KLS cases were secondary and 168 were primary.[7]

## Etiology

An underlying hypothalamic pathology is suggested by the critical role of this structure in regulating sleep, appetite, and sexual behaviors; however, no consistent hypothalamic abnormalities have been identified. Structural brain imaging and evaluation of the cerebrospinal fluid (CSF) and serological inflammatory markers are unremarkable.[7] Electroencephalographic (EEG) slowing is notable in most cases during episodes, without epileptic activity.[7] Diffuse brain hypoperfusion, mostly focused on the thalamic and frontotemporal areas, has been reported.[12] Viral and autoimmune causative factors have been suggested, on the basis of the frequent report of flu-like symptoms at onset, and the most frequent precipitating factor (70%).[13] In few case reports, inflammatory lesions in the thalamus, diencephalon, and midbrain in postmortem neuropathology, suggesting a viral infection.[14,15] Dauvilliers *et al*. found an increased frequency of the human leukocyte antigen DQB1 * 0201 allele.[14] In few cases, abnormalities in serotonin and dopamine metabolism have been reported, suggesting a neurotransmitter imbalance in the serotonergic or dopaminergic pathway.[16,17] The eventual, spontaneous disappearance of the syndrome is as mysterious as the mechanisms determining its periodicity, and should prompt future investigation.

## Precipitating Factors

The first episode of KLS occurred most often in autumn (31.1%) or winter (31.1%), peaking in December (14.8%). Eighty-nine percent of patients remembered an event closely associated with onset, most often infections (72%; 25% with a cold-like syndrome with fever), alcohol use (23%), sleep deprivation (22%), unusual stress (20%), physical exertion (19%), travelling (10%), head trauma (9%), and marijuana use (6%).[7]

## Clinical Presentation

### Hypersomnia

Hypersomnia, a major clinical symptom of KLS, is mandatory for diagnosis and was present in all cases. When reported, usual sleep duration during episodes ranged from 12 to 24 h/day (mean: 18.62 h, median: 18 h).[7] Prodromic symptoms included sudden overwhelming tiredness, i.e. “feeling drawn toward bed,” or “reluctant to get up in the morning.”[18] Several authors noted that their patients remained arousable, waking up spontaneously to void and eat, but were irritable or aggressive when awakened or prevented from sleep. The need for sleep was very intense and at the end of an episode, a short-lasting insomnia was noted in few cases.[19,20] Sleep symptoms changed from frank hypersomnia during the first episodes to a heavy fatigue accompanied by a feeling “as if in twilight between sleep and waking” during later episodes.

## Cognitive Disturbances

Majority of patients had cognitive disturbances such as confusion, concentration, attention, and memory defects. These were evident when interviewed during episodes (such as abnormal responses to question), or reported on subsequent interviews, as a recall of a previous episode.[7]

Abnormal speech has been reported in two-thirds of cases. This included being mute, without spontaneous speech, using monosyllabic or short sentences with limited vocabulary, having slurred, muddled, incoherent, or childish stereotypical language, slow to speak and to comprehend, with verbal perseverations (such as answering with the time at each question), or echoing questions. Many patients reported amnesia of the events that occurred during an attack. Between episodes, majority of patients were described as totally normal. In a few cases reported, academic decline and a mild, long-lasting memory dysfunction between episodes.[21,22] The possibility of residual dysfunction after KLS termination was also reported.[23,24] Temporal disorientation was twice as frequent as spatial disorientation. Altered perception was always present and could affect all senses, with feelings that things were unreal, dreamlike.

## Eating Behavior Disorders

Three quarters of the patients had changes in eating behaviors during episodes. The majority typically ate larger amounts of food (megaphagia) with a preference for sweets and atypical food choices. Patients tended to eat any and all food that was presented. Increased food intake ranged from a mild increase to “three times his usual diet” or “6–8 meals a day” with a 7–30 lb (3.2–13.6 kg) weight gain.[25] Increased drinking of water and juice was also occasionally present, but was never observed alone. A minority of patients had an aversion to food or ate less during one or several episodes, but would overeat during other episodes.[26] Food cravings and megaphagia were the most critical elements.

## Mood Disorders and Irritability

Half of the patients had a depressive mood during episodes particularly in women. Around 15% of the patients reported suicidal thoughts and two patients attempted suicide.[7] In most cases, the depressed mood resolved at the end of each episode, although in rare cases it persisted longer. A few cases reported to be hypomanic for a couple of days at the end of a KLS episode.[27,28] A few patients had a flattened affect, and few were anxious, and panicking when left alone. Irritability was present in almost all patients, especially when sleep, sexual, or food drive were prohibited. It culminated in rare, but severe aggressive behavior. All these abnormalities were transient and reversed after episodes.

## Hypersexuality and Other Compulsive Behaviors

Nearly half of the patients had symptoms consistent with hypersexuality during episodes, and it was significantly more frequent in men than in women. In males, these included increased/overt masturbation, exposing oneself, obscene language, fondling genitalia, and making unwanted sexual advances. Inappropriate sexual advances included the assaulting of female nursing staff, female visitors, patient’s sisters, daughter or other female relatives, and in three cases another man.[7,29,30] Other compulsions that occurred during the episodes included inappropriate and compulsive singing, body rocking, chewing lips, compulsive writing on walls and stripping down wallpaper, and the compulsion to set fire.

## Derealization, Hallucination, and Delusion

A feeling of unreality (surroundings seemed wrong, distorted or unreal, as in a dream) or of disconnected thinking during episodes was reported by most patients and felt to be the most specific symptom of the syndrome. Altered perception was expressed qualitatively as feeling “strange,” “detached,” or “different.”[31] Objects were perceived to be a long way off and voices to be distant with an “unpleasant perception, bizarre, and wrong,” “with a nightmarish sense of the surroundings,” or “with the feeling of being almost in a dream” depersonalization, anguish, and “a persistent sense of unreality and disconnection” from the environment.[11,22] Aside from this feeling of unreality, some patients experienced visual or auditory hallucinations and paranoid or paranoiac delusions.

## Personal and Family Medical History

Birth history in few individuals revealed long labor, hypoxia, premature, or postmature birth. In few cases, there was an impaired development history in the form delayed speech, walking, or reading. Few subjects had been treated for attention-deficit hyperactivity disorder before KLS. There was no increased frequency of psychiatric, neurodegenerative, genetic, or autoimmune diseases in the first-degree relatives of KLS patients compared with control subjects [Table 1].[32]

**Table 1:** Frequency of symptoms during episodes of Kleine–Levin syndrome[7,36]

Symptoms	Percentage (range)
Hypersomnia	100
Cognitive disorders	96–100
Abnormalspeech	60–94
Confusion	51–91
Amnesia	48–66
Hallucinations	14–27
Delusions	16–35
Eating behavior disorders	80–95
Megaphagia	62–66
Increased drinking	6.4–16
Hypersexuality	48–53
Increased masturbation	29
Unwanted sexual advances	17
Irritability	65–92
Depression	48–53
Meningeal and autonomic symptoms	6–89

## Restoration of Normal Function Between Episodes

Arnulf *et al*. compared 108 patients, 79 parent pairs, and 108 matched control between episode about behavior, biological marker and found to be patients and control subjects were remarkably similar between episodes. Despite having similar eating habits and a low frequency of bulimia, patients had a higher body mass index (BMI) *versus* control subjects regardless of time spent in bed. Patients also had increased leptin, C--reactive protein, and greater frequency of snoring and witnessed apnea, but these disappeared after adjusting for BMI. Of interest, BMI did not differ between patients with and without megaphagia. Habitual sleep and wake time were similar in cases and control subjects between episodes. Patients were slightly more anxious than control subjects. There was no difference in mean depression scores between groups.[32]

## Diagnosis

Diagnosis of KLS is very difficult since there are no symptoms that allow for a positive diagnosis. KLS is instead a diagnosis of exclusion, where a doctor must first eliminate a long list of other conditions that could mimic the symptoms. The diagnosis is entirely clinical. According to the International Classification of Sleep Disorders, it belongs to the category of recurrent hypersomnia, defined as episodes of excessive sleepiness lasting more than 2 days and less than 4 weeks, intermixed with long intervals of normal alertness lasting usually months to years, recurring at least every year, and not better explained by a sleep disorder; a neurologic disorder (e.g., idiopathic recurrent stupor, epilepsy); a mental disorder (e.g., bipolar disorder, psychiatric hypersomnia, depression); or the use of drugs (e.g., benzodiazepines, alcohol). The essential clinical criterion of KLS is recurrent episodes of hypersomnia. Moreover, patients have to experience at least one of these symptoms only during the episodes: (1) cognitive or mood disturbances (confusion, irritability, mutism, aggressiveness, derealization, hallucinations, and delusion), which is almost always present; (2) megaphagia with compulsive eating; (3) hypersexuality with inappropriate or odd behavior; and (4) abnormal behavior such as irritability, aggression, and odd behavior.

## Differential Diagnosis

People with KLS are often mistakenly diagnosed with a psychiatric disorder. The periods of somnolence, hyperphagia, and withdrawal can mimic severe depression, and some people experience a brief period of high energy following these episodes which looks like a manic episode, so that some patients are incorrectly diagnosed with bipolar disorder. There can also be a number of other mood symptoms or perceptual disturbances which mimic primary psychiatric disorders.

Narcolepsy, Klüver-Bucy syndrome, and temporal lobe epilepsy (which was ruled out here by EEG) can also produce similar symptom profiles. Multiple sclerosis also has neurological components that can mimic the symptom profile for KLS.

Idiopathic recurrent stupor caused by benzodiazepine or endodiazepine (episodes are shorter and not associated with derealization), metabolic encephalopathies, for example, those with hyperammonemia such as mild cases of ornithine transcarbamylase deficiency (but patients present with protein intolerance, intense vomiting, and gastrointestinal symptoms not seen in KLS; further, the EEG is also abnormal). Before a final diagnosis can be made, all other possibilities must be carefully excluded, and the cluster of symptoms must fit with those commonly observed in KLS patients.

## Investigation

Clinical examination was unremarkable in all cases with primary KLS. In particular, the absence of neurological signs indicative of a focal lesion or of meningitis was notable. The medical tests in KLS patients were mainly aimed at eliminating epilepsy (EEG), focal brain lesions (brain imaging), and meningitis or encephalitis (CSF analysis) as potential causes.

## Cerebrospinal Fluid Analysis

CSF analysis is done when infectious etiology was the possible cause for recurrent hypersomnia. In few cases, CSF examination was done to look for biological changes in CSF and to look for underlying etiology of KLS and found varying results. The serotonin and a serotonin metabolite were increased in one patient, and levels of hypocretin-1, a hypothalamic peptide that has been shown to be deficient in narcolepsy, were found within normal ranges in five KLS patients but slightly decreased in two patients during an episode.[17,32,33,37]

## Electroencephalograms and Brain Imaging

Three-fourth of the patients had a abnormal EEG during episodes, but this is not conclusive, nonspecific and by this we can barely rule-out possibility of epilepsy. The polysomnographic (PSG) studies performed on 17 patients during early part of illness (before the end of the first half of the symptomatic period), an important reduction in slow wave sleep (SWS) was always present with progressive return to normal during the second half despite persistence of clinical symptoms. Rapid eye movement (REM) sleep remained normal in the first half of the episode, but decreased in the second half: the differences between the first and second half of episodes were significant for SWS and REM sleep.[31] However, majority of studies report in 70% of the patients, a nonspecific diffuse slowing of background EEG activity, such as the alpha frequency band being slowed toward 7–8 Hz, was observed. Less often, low-frequency high-amplitude waves (delta or theta) occurred in isolation or in sequence, mainly in the bilateral temporal or temporofrontal areas. A remarkable finding was the ubiquitous absence of epileptic activity. Rarely, isolated spike discharges, self-limited photoparoxysmal response or sharp waves were observed, but were considered of no clinical significance.[32]

Brain computerized tomography and magnetic resonance imaging were normal in all cases. Functional imaging measuring cerebral blood flow by single photoemission tomography was performed in few patients aged 13–27 years showed reduced blood flow in few and normal in few. The reduction occurred in the temporal or temporofrontal areas of either or both sides and in the basal ganglia. In one case report, single photon emission computed tomography (SPECT) was done during episode and interepisodic period, and it reports significant hypoperfusion in the left hypothalamus, bilateral thalami, basal ganglia, bilateral medial and dorsolateral frontal regions, and left temporal lobe during the symptomatic period.[33]

## Hormonal Tests

Changes in levels of pituitary hormones were only rarely found in KLS patients. Thyroid profile, pituitary, adrenal profile, and blood sugar were done mainly to exclude possible endocrinological cause for recurrent hypersomnia.

## Treatment

There is no definitive treatment for Kleine–Levin syndrome during episode as well as interepisodic period. Cochrane review published in 2009 found no randomized, placebo-controlled trials of pharmacological treatments for Kleine–Levin syndrome.[34] Various medications have been used during episode in many case reports and found to be no consistent benefits from any one of the drugs. Various stimulants, including methylphenidate, modafinil, pemoline–piracetam–meclofenoxate, D-amphetamine, ephedrine, methamphetamine, amphetamine, etc., can be used to treat sleepiness, but unfortunately do not improve sluggish cognition or other elements of the altered mental state.[34] However, with amphetamine response rate found to 71% compared to rest of stimulants.[7] The lithium was also tried during episode. Lithium had significantly improved abnormal behavior and recovery of symptoms (reducing the duration of episodes). The response rate with lithium is 41%. In few case reports, it has been shown that good response to lithium and when drug was withdrawn reappearance of symptoms.[24] When lithium was reintroduced, they recovered again. One of the possible causes could be similarities between Kleine–Levin syndrome and bipolar disorder (e.g., recurrence of episodes and sudden changes from a depressed mood during an episode to hypomania at the end of an episode in some patients). Similar kind of benefit was also noticed with carbamazepine in few case reports.[35] Responses to treatment have often been limited, and there is no evidence to support the use of these therapies. Various case reports have used medications such as flumazenil, chlorpromazine, levomepromazine, trifluoperazine, haloperidol, thioridazine, clozapine, and risperidone and found to be ineffective. Electroconvulsive therapy and insulin coma therapy had no effect on KLS symptoms (and even worsened confusion in the case of electroconvulsive therapy).

During interepisodic period, various mood stabilisers, such as lithium, carbamazepine, valproate, phenytoin, and phenobarbital, were tried as mentioned above similarities between bipolar disorder and KLS. Of these treatments, only lithium had a reported response rate significantly higher than medical abstention. It has reduced chances of relapses.[7,35] Even carbamazepine has also reduced number of relapse in many case reports; however, there are no consistent benefits due to this medication and the clinicians has weigh risk and benefits of both these medications. In future, we may need to work on double-blind placebo-controlled therapeutic trials of drugs for both during episode as well as interepisodic period. We also need to try medications such as immune suppressants as illness could be due to immune-related disorder and also antivirals as its onset usually follows viral infection in majority of subjects [Table 2].

**Table 2:** Treatments used in patients with Kleine–Levin syndrome and reported effects[7,36]

Treatments of symptoms during an episode	Response rate (%)
Stimulants (reduction of hypersomnia)	
Amphetamines	40–71
Methylphenidate	20
Pemoline–piracetam–meclofenoxate	25
Treatments aimed at preventing relapses	
No drug treatment	16
Phototherapy	0
Antidepressants	9
Mood stabilizers	
Lithium	41
Carbamazepine	21
Valproate, phenobarbital, and phenytoin	20
Various	
Antiviral (i.v. acyclovir)	0
Melatonin	0
Benzodiazepines	0
Levodopa + benserazide	0
Electroconvulsiv etherapy	0
Neuroleptics	0

## Course and Prognosis

Kleine–Levin syndrome has a benign clinical course, with spontaneous disappearance of symptoms. A 2005 study of 186 KLS patients reported that in subjects where the disease terminates, the average age is 23 and the median duration is 4 years. They reported no correlation between age at onset and disease duration. Patients experienced an average of 12 episodes lasting an average of 12 days, although the range of symptoms reported varied from 2 to 130 episodes and lasted between 2.5 and 80 days. Subjects experienced an average duration of 6 months between episodes, but this ranged from 0.5 to 72 months. Subjects typically experienced less frequent and less intense attacks toward the end of the disease course, and the subject is considered cured if they do not experience an episode for 6 or more years. The median disease duration is 10 years in patients without hypersexuality, but 21 years in patients with hypersexuality. The duration also appears to be more years for patients initially struck as adults.[7]

Women had a longer disease course than men, despite a comparable age at KLS onset and an absence of differences in the duration of episodes and symptoms-free intervals. Women had the same frequency of megaphagia and psychotic symptoms, but a lower frequency of hypersexuality and cognitive impairment. Patients with a high number of episodes during the first year of KLS had a somewhat shorter KLS disease duration. In contrast, the age at KLS onset, the presence of megaphagia, cognitive disturbances, psychotic signs, and hypersexuality did not influence the course of the disease. Most notably, in patients with “full-blown” KLS (suffering from hypersomnia, cognitive disturbances, eating disorders, and hypersexuality) did not have different disease duration when compared to 63 patients with “incomplete” KLS.[7]

## Conclusion

KLS is an intriguing, severe, homogenous disease, known for more than a century, with defined clinical features, but no clear cause or treatment. Therefore, for studies on KLS was done exclusively with case reports or case series. There are limited systemic study on comparing well-defined KLS with control group in terms of phenomenology, biological cause, and lists of investigations to identify disease and its management. However, few study findings suggest the possible Jewish predisposition, occasional familial clustering, and the association with infectious triggering factors suggesting that KLS is caused by environmental factors acting on a vulnerable genetic background. Recent methods of radiological investigation, such as SPECT, indicate that the brain dysfunction could be larger than expected, and encompass both cortical and subcortical (and especially thalamus and hypothalamus) areas. This general picture and the fluctuating symptomatology in KLS are consistent with the recent report of an HLA association in KLS and the possibility of an autoimmune mediation of the disorder. Due to rarity of disorder, it is difficult to identify underlying biological cause. In future, we need further research on genetic etiology and management of this disorder.
